# Evaluating Ribosomal Protein L19 mRNA as a Biomarker in Ulcerative Colitis: Implications for Severity Assessment

**DOI:** 10.1155/mi/4823996

**Published:** 2026-04-25

**Authors:** Lihao Shi, Guiyuan Jin, Xizhuang Gao, Yun Chen, Qian Zhao, Jian Lin, Guangxi Zhou

**Affiliations:** ^1^ Department of Gastroenterology, Affiliated Hospital of Jining Medical University, Jining, Shandong, China, jnmc.edu.cn; ^2^ Medical Research Center, Affiliated Hospital of Jining Medical University, Jining, Shandong, China, jnmc.edu.cn; ^3^ Department of Functional Examination, Zaozhuang Municipal Hospital, Zaozhuang, Shandong, China, zzslyy.com; ^4^ Department of Gastroenterology, Affiliated Hospital of Putian University, Putian, Fujian, China, ptu.edu.cn

**Keywords:** biomarkers, disease severity, inflammatory bowel disease (IBD), RPL19 mRNA

## Abstract

**Background:**

Ulcerative colitis (UC) is a chronic inflammatory disease of the gastrointestinal tract, characterized by immune dysregulation, genetic susceptibility, and environmental factors such as diet and psychosocial stress. The etiology of UC is complex, involving multiple interrelated factors that drive its pathogenesis and clinical progression.

**Methods:**

This cross‐sectional study investigated the potential role of ribosomal protein L19 (RPL19) mRNA in UC. A total of 40 patients with UC and 29 healthy controls (HC) were prospectively enrolled from the Department of Gastroenterology, Affiliated Hospital of Jining Medical University, between November 2021 and November 2023. RPL19 mRNA levels in the intestinal mucosa of UC patients were quantified using quantitative PCR analysis. Subsequently, the associations of RPL19 mRNA expression with disease severity and with the levels of key inflammatory cytokines were statistically assessed.

**Results:**

RPL19 mRNA expressions were significantly regulated in UC patients compared to HC. It exhibited a significant inverse correlation with the levels of both interleukin‐2 (IL‐2) and interleukin‐4 (IL‐4). Importantly, RPL19 mRNA levels also correlated with key clinical disease activity indices.

**Conclusions:**

This study demonstrates that mucosal RPL19 mRNA expression levels are significantly elevated in UC patients and correlate positively with endoscopic and histological disease severity. These findings identify RPL19 as a potential biomarker for reflecting local disease activity in UC.

## 1. Introduction

Ulcerative colitis (UC) is a lifelong inflammatory disease of the gastrointestinal tract. Its pathogenesis involves complex interactions among abnormal immune responses, genetic susceptibility, the gut microbiome, and the environmental risk factors [1–3]. In current clinical practice, disease activity in UC is assessed using endoscopic scoring [4], inflammatory markers, and imaging techniques. However, the clinical application of biomarkers is limited due to their variability in expression, lack of specificity, or quantification challenges [5]. The British Society of Gastroenterology’s 2022 guidelines reaffirm the importance of developing new biomarkers for UC to enhance disease activity assessment and cancer risk prediction [6]. Therefore, the identification of reliable biomarkers holds significant potential to refine clinical management, advance research, and ultimately improve patient outcomes. Ribosomal protein L19 (RPL19) is a component of the ribosomal 60S subunit, which is required for protein synthesis, and belongs to a family of ribosomal proteins essential for cellular function [7–9]. Recently, numerous studies have confirmed its potential function as a cancer biomarker [10], while its role in UC remains largely unknown. In the case of UC, several key points are noteworthy [11]: dysregulated expression of ribosomal proteins, including RPL19, may affect inflammatory pathways, and immune response [12] and inflammation are central to the pathophysiology of UC. Moreover, upregulated inflammatory cytokines in UC will in turn affect the expression of ribosomal proteins, which in turn affects the entire protein synthesis machinery and cellular function [13]. It may even be associated with disruption of apoptosis or survival pathways [14], leading to tissue damage and disease progression in the context of UC.

Therefore, this study aimed to investigate the relationship between RPL19 mRNA expression levels and disease activity in the intestinal mucosa of UC patients, to evaluate its potential as a novel biomarker.

## 2. Materials and Methods

### 2.1. Patient Samples

This was a cross‐sectional study in which 40 voluntary patients with UC were prospectively recruited between November 2021 and November 2023 in the Department of Gastroenterology, Affiliated Hospital of Jining Medical University. Twenty‐nine healthy controls (HC) were also included. The colonoscopy was derived from the patient’s regular review or first diagnosis. HC colonoscopy was derived from the colorectal screening program. The HC group comprised individuals undergoing screening colonoscopy at a health examination center. All participants voluntarily enrolled in this study and signed informed consent forms. All control subjects demonstrated normal colonoscopy findings, with no macroscopic or microscopic evidence of inflammation or other significant pathological changes. The inclusion criteria of the UC group were as follows: (1) diagnosis of UC based on clinical presentation, imaging, endoscopic, and histological criteria; (2) age greater than 18 years; and (3) review and completion of a medical questionnaire by an experienced clinician to collect demographic and clinical information. The exclusion criteria of the UC group were as follows: (1) coexistence of other significant autoimmune diseases, such as systemic lupus erythematosus, rheumatoid arthritis, or asthma, which could confound the assessment of UC; (2) a history of malignancy or serious infection, which could interfere with the evaluation of UC disease activity or the interpretation of RPL19 mRNA levels. Disease activity was determined using several condition assessment scoring tools commonly used in UC, including the Mayo score [15], Dublin score [16], Mayo endoscopic score (MES) [17], and ulcerative colitis endoscopic index of severity (UCEIS) [16]. Disease site and behavior were determined according to the Montreal classification [18]. To assess RPL19 mRNA, C‐reactive protein (CRP), interleukin‐2 (IL‐2), interleukin‐4 (IL‐4), interleukin‐6, interleukin‐17 A, interferon‐γ, and tumor necrosis factor‐*α*, we selected mucosal tissues from the most inflamed sites of colonoscopy for the experiments, and two pieces of each sample were taken to reduce experimental error.

### 2.2. Laboratory Test

The CRP level was measured by rate turbidimetry using Beijing Kangshirunye Biotechnology Co., Ltd. and the serum interleukin‐2, interleukin‐4, interleukin‐6, interleukin‐17 A, interferon‐*γ*, and tumor necrosis factor‐*α* levels were measured by enzyme‐linked immunosorbent assay using the RapidbioLab kit from the United States of America.

### 2.3. Ethics Statement

This study was approved by the Ethics Committee of the Affiliated Hospital of Jining Medical University. All UC patients and HC subjects provided written informed consent in accordance with relevant guidelines and regulations.

### 2.4. Extraction of Total RNA From Intestinal Mucosal Tissue

The intestinal mucosal tissue was placed into the EP tube, and 1 mL of Trizol was added. Repeated grinding with a tissue grinder. The tissue fluid was then transferred to a new EP tube and mixed with 200 *μ*L of chloroform. After 15 min on the ice, centrifuge. The supernatant was transferred to a new EP tube, mixed with the same volume of isopropyl alcohol, and stored in a refrigerator at −20°C for 1–2 h. After centrifugation again, discard the supernatant, add 1 mL anhydrous ethanol, gently blow the precipitation, centrifuge again, and discard the supernatant. After precipitation and drying, DEPC water was added and mixed evenly, and then RNA concentration was measured. The required mRNA 260/280 ratio should be between 1.8 and 2.0.

### 2.5. Quantitative Reverse Transcription‐Polymerase Chain Reaction (qRT‐PCR)

According to the guidelines provided by the manufacturer, we selected 5 × All‐in‐one RT mastermix for RT‐PCR reaction to synthesize complementary DNA. The conditions for RT‐PCR include a first annealing at 25° C for 10 min, a reverse transcription process at 42° C for 15 min, and a thermal activation step at 85° C for 5 min. After obtaining cDNA, we selected the SYBR Green PCR kit for qRT‐PCR analysis of gene transcription level. Follow the instructions (95° C for 1 min, then 40 cycles at 95° C for 15 s, 60° C for 30 s for 45 cycles). As an endogenous reference gene, GAPDH is primed in the following Table 1. To calculate the relative expression of target gene expression, qRT‐PCR analysis was performed using 2−ΔΔCt based on the ratio relative to GAPDH.

**Table 1:** Sequences of primers used for quantitative reverse transcription–polymerase chain.

Gene	Primer sequence	Length
GAPDH	Forward: GGAGCCAAAAGGGTCATCATCT	22
	Reverse: GAGGAGCCATCCACAGTCTTCT	22
RPL19	Forward: AAAACAAGCGGATTCTCATGGA	22
	Reverse: TGCGTGCTTCCTTGGTCTTAG	21

### 2.6. Disease Score and Severity Rating

According to the modified Mayo score, the experimental group can be divided into four groups: clinical remission group (score ≤2), mild group (score 3–5), moderate group (score 6–10), and severe group (score 11–12). According to the MES score, endoscopic findings can be classified into four grades. The MES1 score defines the mild group. MES2 score belongs to the moderate group. MES3 score to the severe group. MES3 score to the severe group. MES3 score to the moderate group. The UCEIS score classifies patients into remission group (0–1), mild group (2–4), moderate group (5–6), and severe group (7–8) depending on the score. Based on the Dublin score, patients were assessed for endoscopic disease activity, which was classified into different grades according to the scores: <3, 3~4, 6 and 9. The degree of disease inflammation in specific patients with different scores is detailed in Table 2.

**Table 2:** Laboratory indices and disease activity scores in patients with ulcerative colitis.

Parameter	UC (*n* = 40)
CRP (mg/L)	9.66(0.23–54.23)
MES	
1	3
2	18
3	19
UCEIS	
2–4	16
5–6	19
7–8	5
Mayo	
3–5	12
6–10	22
11–12	6
Dublin	
<3	8
3–4	11
6	9
9	12

### 2.7. Statistical Analysis

Results are reported as mean ± standard deviation, test in triplicate. Unpaired*t*‐test or Mann–Whitney *U* test were used to compare the continuous variables between the two groups. Multiple comparisons were made using ANOVA or *χ*2 test. Pearson analysis was used to determine the linear correlation between different groups. The clinical diagnostic value of RPL19 mRNA was evaluated by characterization curve analysis. Risk factors were determined by logistic regression, and a *p*‐value less than 0.05 was considered statistically significant. SPSS 19.0 (SPSS Inc.) was used for statistical analysis.

## 3. Result

### 3.1. Characteristics

The demographic and clinical data for the UC patients and HC are presented in Table 3. The data include 40 UC patients, consisting of 27 males (67.5%) with a mean age of 49.83 years, and 29 HC, comprising 21 males (72.4%) with a mean age of 56.69 years. The age (*p*  = 0.3174) and sex (*p*  = 0.661) distributions were not significantly different between the groups. The location of onset of disease in the patients was E1(11), E2(11), and E3(11). According to the MES score, patients were categorized as mild, moderate, and severe (3,18, and 19), respectively, while UCEIS (16,19,5), Mayo (12,22,6), and Dublin (8,11,9,12) were used.

**Table 3:** Clinical characteristics of patients and controls.

Characteristic	UC (*n* = 40)	HC (*n* = 29)	*p*‐Value
Male/female	27/13	21/8	0.661
Mean age (year)	49.83 ± 15.18	56.69 ± 12.65	0.3174
Range (year)	17–78	26–74	—
Tobacco smoking (*n*)			0.705
Never	21	16	—
Past or current use	19	13	
Disease duration (year)	2.50 ± 3.80	—	
Disease location (*n*)	—		
E1	11		
E2	11		
E3	18		

### 3.2. RPL19 mRNA Expression Was Increased in the Intestinal Mucosa of UC Patients

We initially assessed whether RPL19 mRNA expression conformed to a normal distribution in HC and UC patients (Figure 1A). Subsequent analysis revealed that compared to HCs, RPL19 mRNA expression in the intestinal mucosa was significantly increased in UC patients (Figure 1B). There was no significant difference in RPL19 mRNA expression between UC patients with a smoking history and non‐smokers (Figure 1C). RPL19 mRNA demonstrates diagnostic value in distinguishing between UC and non‐UC groups. The assay for RPL19 mRNA in the intestinal mucosa has a sensitivity of 0.5, indicating its limited reliability as a standalone diagnostic tool for UC. However, its specificity of 0.897 is noteworthy, as it effectively identifies individuals without the disease. This high specificity is particularly valuable, suggesting that negative assay results can reliably rule out UC, potentially avoiding unnecessary further testing in people who do not have the disease. According to our findings, when the RPL19 mRNA threshold exceeds 2.108, the test result is considered positive for UC (Table 4).

Figure 1Relative expression of RPL19 mRNA in UC patients and HCs. RPL mRNA was elevated in patients with UC compared with HC. (A) Normality of RPL mRNA expression in patients with HC and UC. (B) The expression of RPL mRNA was different between the HC group and the UC group, and the difference between the two groups was determined by an unpaired *t*‐test. (C) There was no difference in the expression of RPL mRNA between UC patients with smoking history and those who had never smoked. The difference between the two groups was determined by unpaired *T*‐test. Student’s *t*‐test:  ^∗^ *p* < 0.05, ns, not significant.

**Figure:**
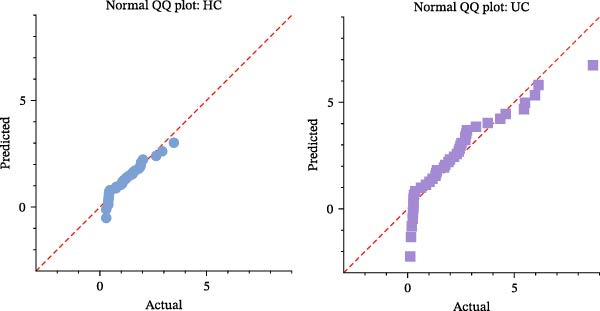
(A)

**Figure:**
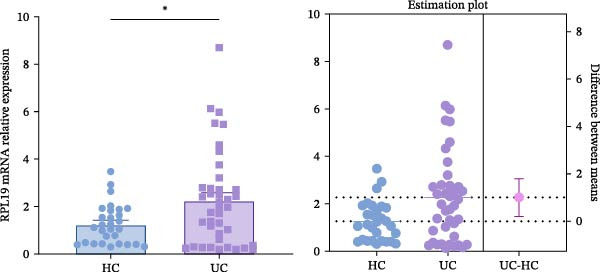
(B)

**Figure:**
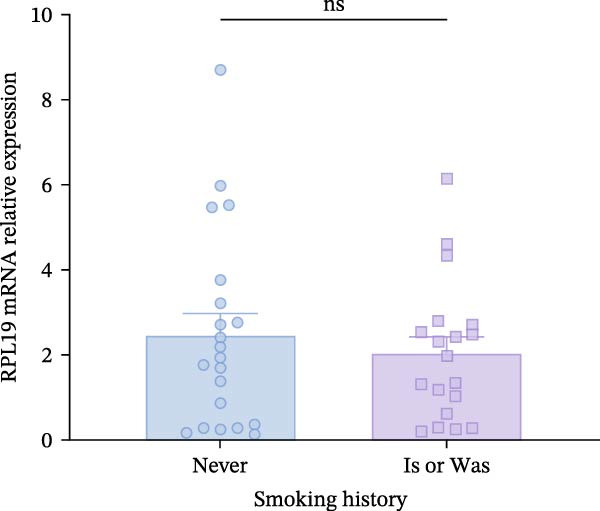
(C)

**Table 4:** Receptor operator characterization of RPL mRNA in the intestinal mucosa of patients with ulcerative colitis.

Group	AUC (95% CI)	*p*‐Value	Cutoff value	Sensitivity	Specificity	PPV	NPV	LR+	LR−	Diagnostic accuracy
UC vs HC	0.646(0.511–0.781)	<0.05	2.108	0.500	0.897	0.863	0.553	4.591	0.586	0.533

### 3.3. RPL19 mRNA Expression Was Positively Correlated With Disease Severity in UC Patients

Although we found that RPL19 mRNA was not entirely satisfactory in accurately identifying all cases of UC at an early stage, its high specificity markedly reduces the number of false‐positive cases. We still believe it holds potential as a valuable tool in the diagnostic process, especially when used alongside other diagnostic tests and clinical evaluations. Accordingly, we scored 40 patients with colitis for disease severity. Using the Montreal classification, we analyzed RPL19 mRNA expression in intestinal mucosal tissue across three disease extent groups: E1, E2, and E3. The differences in expression among these groups were not statistically significant (Figure 2A). In contrast, the expression level of RPL12 mRNA was significantly higher in the intestinal mucosa of patients with severe UC. In terms of the UCEIS, the intestinal mucosal RPL19 mRNA expression in the severe group was significantly higher than in the moderate and mild groups (*p* < 0.001). Additionally, the expression difference between the moderate and the mild groups was also statistically significant (*p* = 0.0127; Figure 2B). Furthermore, within the MES groups, the expression level of intestinal mucosal RPL19 mRNA in the severe group was significantly higher than in the mild (*p* = 0.0297) and moderate groups (*p* = 0.0157), although the difference between the moderate and mild groups was not statistically significant (*p* = 0.5124; Figure 2C). As for the Mayo score, for the intestinal mucosal tissues of UC patients in the severe group, the expression level of RPL19 mRNA was significantly increased (*p* = 0.0022). However, there was no statistically significant difference in RPL19 mRNA expression between the mild and moderate groups (Figure 2D).

Figure 2Comparison of RPL19 mRNA expression in the intestinal mucosa in patients with different disease sites and different disease severities. (A) The expression of RPL19 mRNA in the intestinal mucosal tissues of three groups, E1, E2, and E3, was compared according to Montreal typing. (B) In the UCEIS score subgroups, the expression level of RPL19 mRNA in the intestinal mucosal tissues of the patients with UC. (C) In the MES score group, the expression level of RPL19 mRNA in the intestinal mucosal tissues of the patients with UC. (D) In the Mayo score, the expression level of RPL19 mRNA in the intestinal mucosal tissues of the patients with UC. Student’s *t*‐test:  ^∗^ *p* < 0.05,  ^∗∗^ *p* < 0.01,  ^∗∗∗^ *p* < 0.001, ns, not significant.

**Figure:**
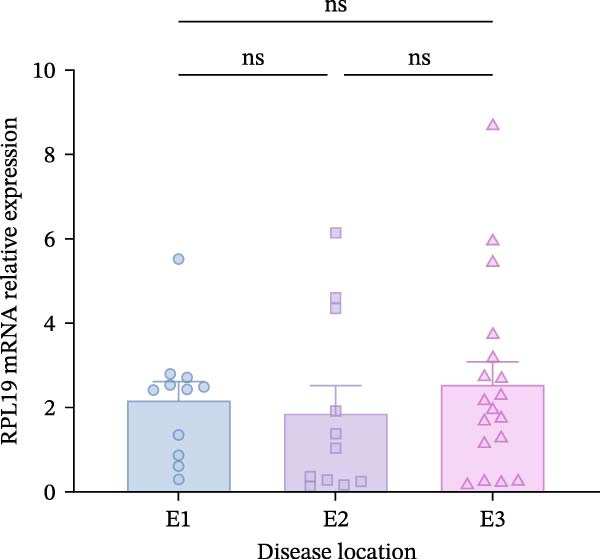
(A)

**Figure:**
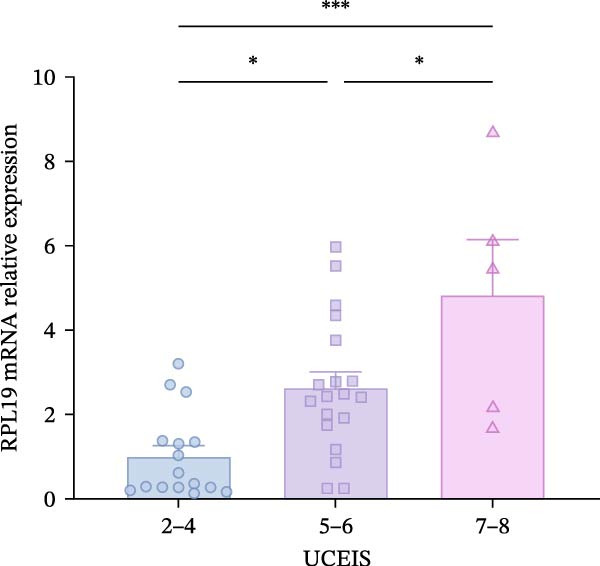
(B)

**Figure:**
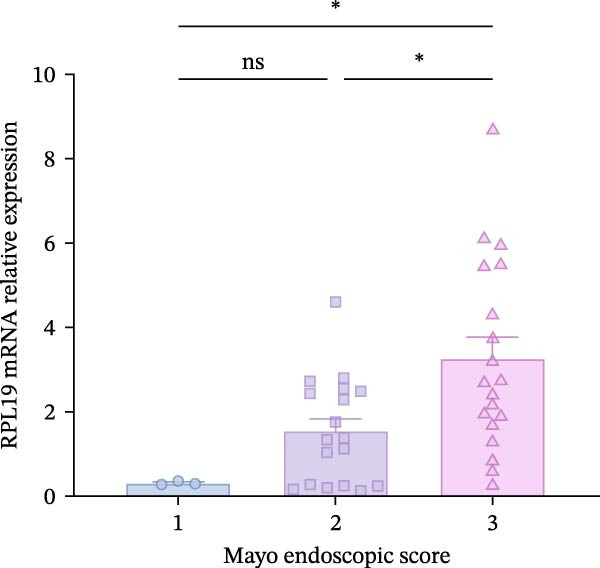
(C)

**Figure:**
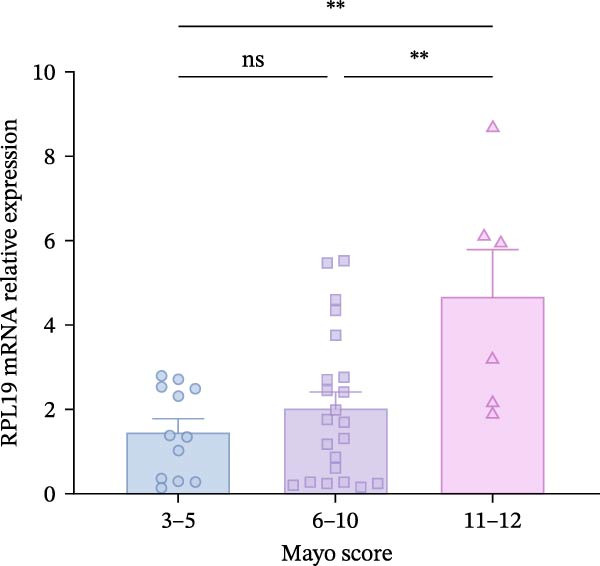
(D)

We performed a statistical analysis to explore the correlation between RPL19 mRNA expression levels in the intestinal mucosa and commonly used endoscopic scoring systems, including the MES, the UCEIS, Mayo scores, and the Dublin scores (Figure 3A–D). Our analysis revealed a positive correlation between RPL19 mRNA levels and all the endoscopic scores, which was statistically significant. Notably, the UCEIS score showed the strongest correlation with RPL19 mRNA levels (*R* = 0.558, *p* = 0.0002), matching the MES (*R* = 0.4998, *p* = 0.001) and Mayo scores (*R* = 0.486, *p* = 0.0015), with the Dublin score presenting a similarly strong but slightly lower correlation (*R* = 0.347, *p* = 0.0281). The UCEIS is specifically designed to offer a more objective and reproducible assessment of the endoscopic severity of UC, focusing on three key findings: vascular morphology, bleeding, and ulcers. The strong correlation with UCEIS highlights the potential of RPL19 mRNA as a molecular marker of disease severity in UC [19]. Furthermore, the absence of a correlation between RPL19 mRNA expression levels and age suggests that the mechanisms reflected in RPL19 expression are directly related to the disease process rather than age‐related changes in the intestinal mucosa, underscoring its value as a disease‐specific biomarker (Figure 3E).

Figure 3Correlation between RPL19 mRNA expression and MES, UCEIS, Mayo, and Dublin scores in patients with inflammatory bowel disease and age in patients with UC. Pearson analysis was applied to test the correlation of RPL19 mRNA expression with disease activity. (A–D) Correlations of RPL19 mRNA expression with disease activity in patients with MES score, UCEIS, Mayo score, and Dublin score. (E) Correlation of RPL19 mRNA expression levels is independent of age.

**Figure:**
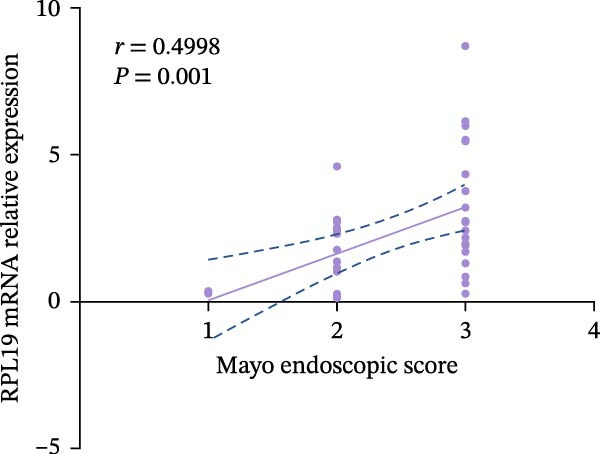
(A)

**Figure:**
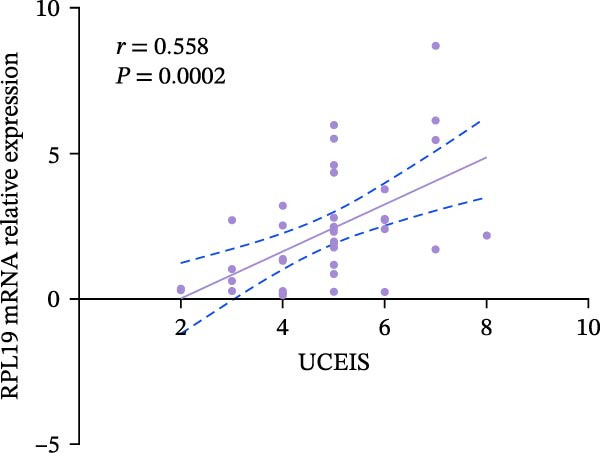
(B)

**Figure:**
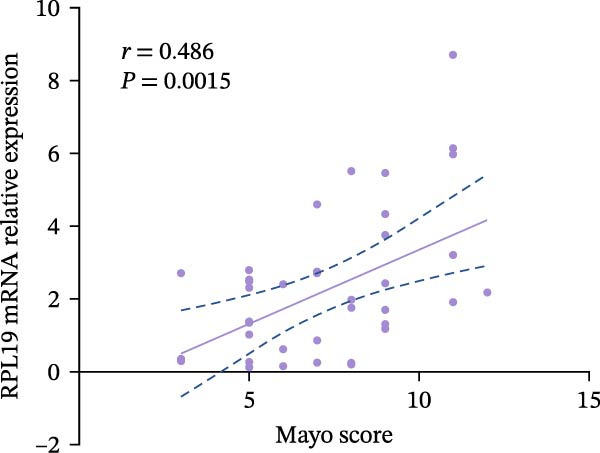
(C)

**Figure:**
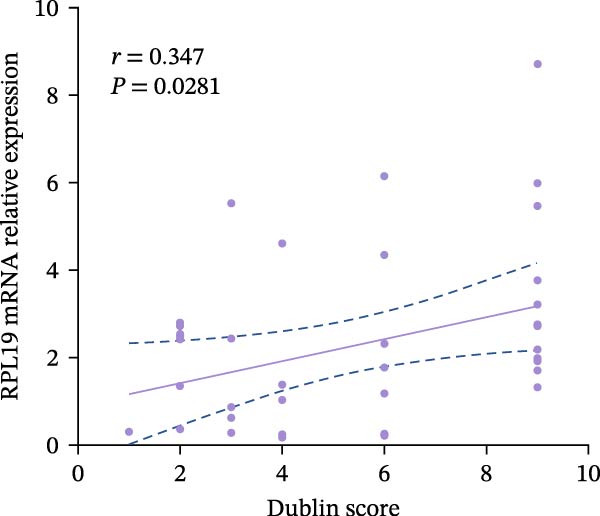
(D)

**Figure:**
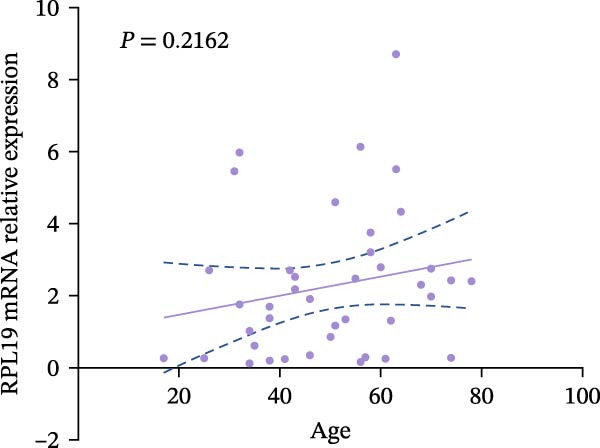
(E)

### 3.4. RPL19 mRNA Expression was Negatively Correlated With Levels of IL‐2 and IL‐4 in UC Patients

In our study, we measured serological inflammatory factor levels in a subset of 13 patients and compared these data to the overall group of 40 patients, finding no statistical differences in the basic data between the two groups (Table 5). We specifically analyzed the expression of inflammatory factors in the sera of these 13 patients; we analyzed interleukin‐2, interleukin‐4, interleukin‐6, interleukin‐17A, interferon‐γ, and tumor necrosis factor‐*α* (Figure 4) and observed that the RPL19 mRNA levels were negatively correlated with IL‐2 and IL‐4 but not statistically correlated with the other inflammatory factors, both of which are key cytokines in the immune system (Figure 4).

Figure 4Correlation of RPL19 mRNA expression with inflammatory cytokines in UC. (A–F) Correlation of RPL19 mRNA expression with interleukin‐2, interleukin‐4, interferon‐γ, interleukin‐6, interleukin‐17 A, and tumor necrosis factor‐*α* in UC. RPL19 mRNA levels were negatively correlated with IL‐2 and IL‐4, but not statistically correlated with the other inflammatory cytokines. Pearson analysis was applied to test the correlation of RPL19 mRNA expression with expression with inflammatory cytokines.

**Figure:**
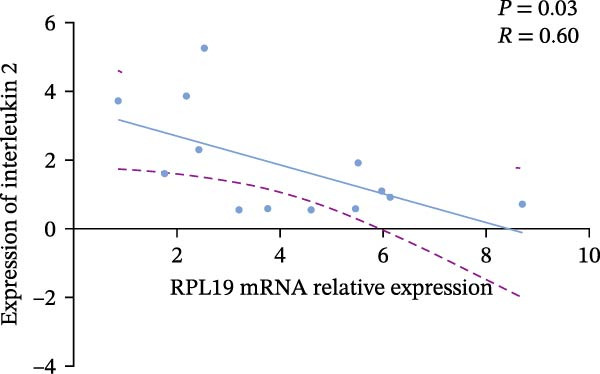
(A)

**Figure:**
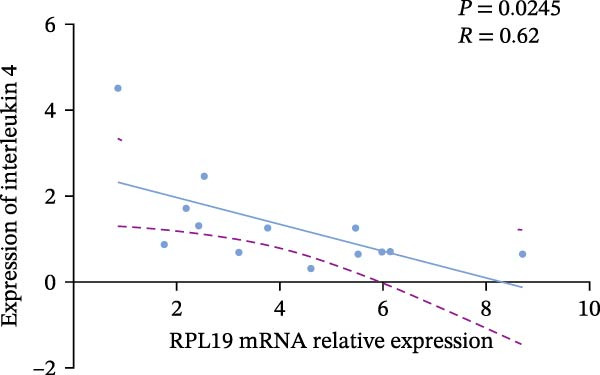
(B)

**Figure:**
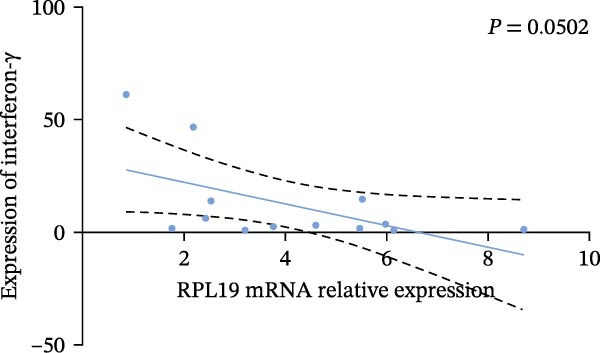
(C)

**Figure:**
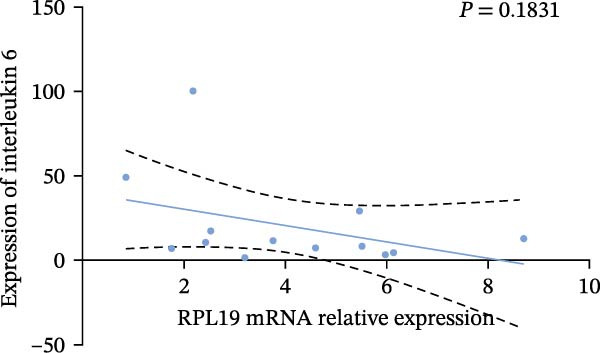
(D)

**Figure:**
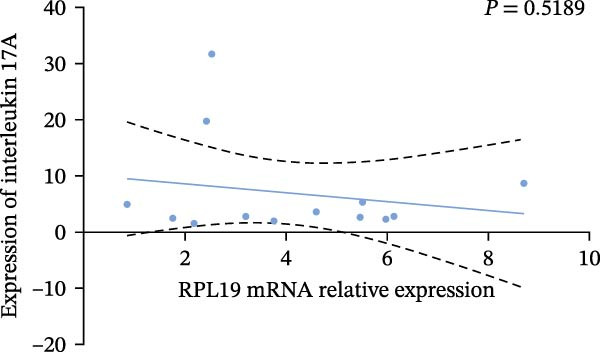
(E)

**Figure:**
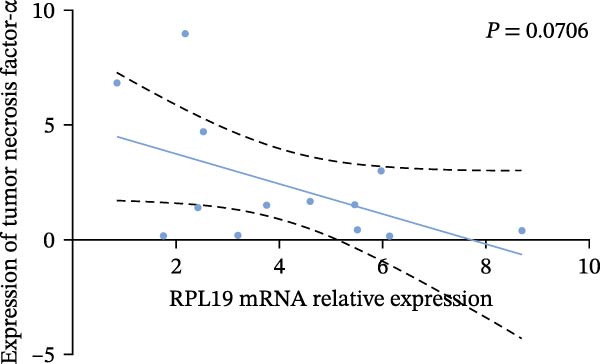
(F)

**Table 5:** Differential analysis between some and overall ulcerative colitis patients.

Characteristic	UC (*n* = 40)	PUC (*n* = 13)	*p*‐Value
Male/Female	27/13	10/3	0.460
Mean age (year)	49.83 ± 15.18	50.31 ± 13.49	0.9715
Range (year)	17–78	31–74	—
Tobacco smoking (*n*)			
Never	21	9	0.3289
Past or current use	19	4	
Disease duration (year)	2.50 ± 3.80	2.74 ± 3.57	

### 3.5. Compared to CRP RPL19 mRNA Expression in the Intestinal Mucosa Enables Better Diagnosis of Patients With Severe Ulcerative Colitis

Receiver operating characteristic (ROC) analysis was conducted to evaluate the diagnostic performance of RPL19 mRNA expression levels in the intestinal mucosa. The analysis revealed that RPL19 mRNA has a superior ability to assess the severity of UC as defined by the modified Mayo score compared to CRP, with an Area under the curve (AUC) value of 0.8333 (*p* = 0.01). In contrast, CRP’s AUC for the same score was only 0.5931 (*p* = 0.4717), indicating less reliability (Figure 5A). Further analyses demonstrated that RPL19 mRNA also outperformed CRP in predicting severe disease as measured by the MES3 score and UCEIS ≥7 score, with AUC values of 0.7744 (*p* = 0.003) and 0.8000 (*p* = 0.0318), respectively. Conversely, the AUC values for CRP were 0.7231 (*p* = 0.0159) and 0.7629 (*p* = 0.0599) for these scores (Figure 5B, C).

Figure 5ROC analysis of RPL19 mRNA expression level in intestinal mucosa and CRP in severe UC assessment. (A) Indicates the ROC analysis of RPL19 mRNA expression level in intestinal mucosa and CRP on the evaluation of the severe Mayo score group with improved clinical disease activity. The AUC value of RPL19 mRNA was 0.8333. The AUC of CRP was 0.5931. (B) Represents the expression level of RPL19 mRNA in intestinal mucosa and the ROC analysis of CRP on the evaluation of the MES score of disease activity under endoscopy. The AUC value of RPL19 mRNA was 0.7744. The AUC of CRP was 0.7231. (C) Represents the expression level of RPL19 mRNA in intestinal mucosa and the ROC analysis of CRP on the evaluation of the UCEIS score group with severe disease activity under endoscopy. The AUC value of RPL19 mRNA was 0.8000. The AUC of CRP was 0.7629. The clinical diagnostic value of RPL19 mRNA was evaluated by characterization curve analysis. Risk factors were determined by logistic regression, and a *p*‐value less than 0.05 was considered statistically significant.

**Figure:**
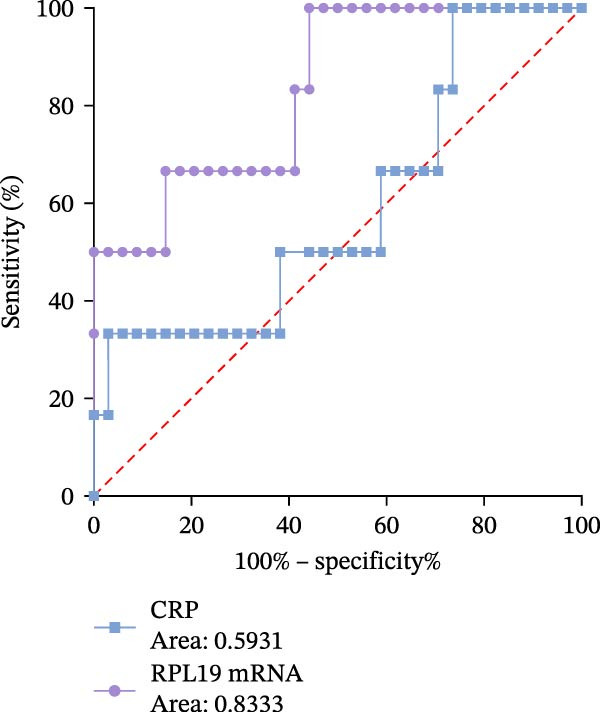
(A)

**Figure:**
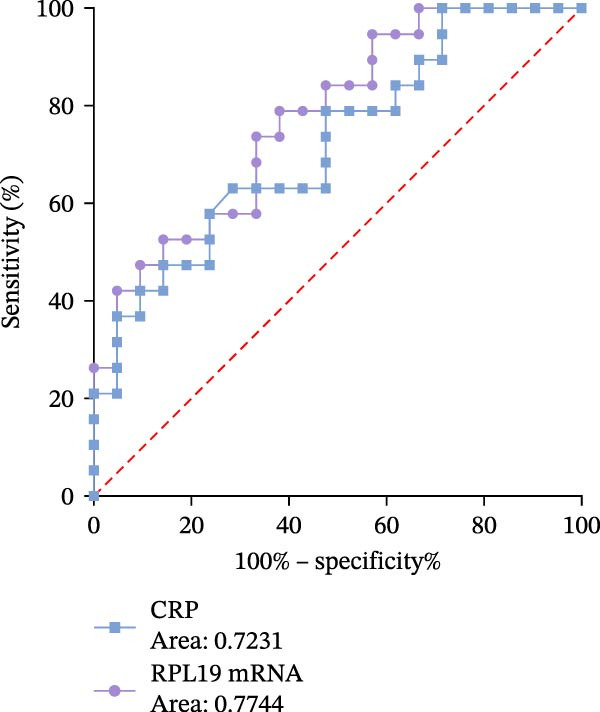
(B)

**Figure:**
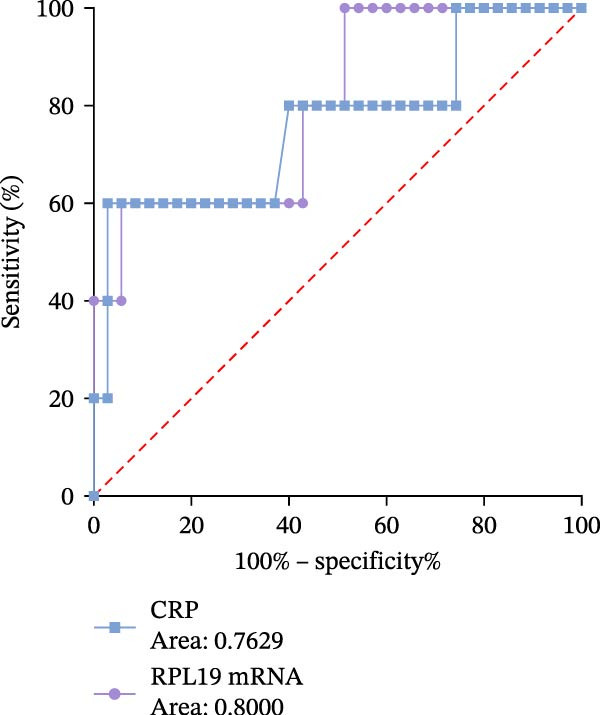
(C)

These results suggest that RPL19 mRNA expression is a potentially more sensitive or specific marker for severe UC compared to CRP, which, despite being a recognized marker of inflammation and widely used in clinical practice, showed less reliability in distinguishing between severe and non‐severe cases of UC. This unreliability might also be influenced by our study’s small sample size. In this research, CRP was utilized primarily as a benchmark to compare the diagnostic performance of newer markers like RPL19 mRNA, emphasizing the innovative introduction of RPL19 mRNA as a potentially superior diagnostic tool for severe UC.

## 4. Disussion

UC is a chronic condition characterized by intermittent periods of intestinal inflammation and remission. The pathophysiological mechanisms underlying this disease are highly complex, involving abnormalities in the immune system, genetic predispositions, environmental influences, and other factors [20, 21]. Inaccurate assessment and ineffective treatment of UC can hinder the healing of the intestinal mucosa, thereby prolonging the disease course. This not only increases the risk of requiring surgical intervention but also elevates the potential for cancer development [20, 22].

In clinical practice, accurate assessment of disease activity in UC is crucial for developing personalized treatment programs, evaluating treatment response, and monitoring for disease recurrence to manage the course of the disease effectively [23]. Colonoscopy is currently considered the gold standard for assessing UC activity. Its capability to observe inflammation directly and accurately in the colon mucosa makes it not only the most effective method for evaluating UC activity but also the best approach for monitoring disease progression [24]. This direct observation provides critical guidance for clinical diagnosis and treatment. Currently, there is no biomarker or method that can completely replace colonoscopy; however, its clinical application is limited due to being an invasive procedure, having high examination costs, and poor repeatability [25]. Although inflammatory indicators and imaging examinations are non‐invasive, have good repeatability, and can timely evaluate the activity of UC, they suffer from poor specificity and low sensitivity, presenting certain limitations in clinical practice [26]. Thus, exploring a biomarker that can accurately and effectively evaluate UC disease activity is of great clinical significance. Previous studies have indicated that RPL19 plays a role in the pathogenesis of autoimmune and chronic inflammatory diseases, and it has been established as a prognostic marker for various tumors [7, 27. Fecal RPL19 expression, related to tumor staging and serum CEA levels, can predict the prognosis of patients with colorectal cancer [8]. However, the relationship between RPL19 and UC disease activity has not been extensively studied. In this research, we conducted an in‐depth analysis of this relationship and discovered that RPL19 mRNA expression in the intestinal mucosa is closely related to UC disease activity. This suggests that RPL19 mRNA could serve as a new biomarker to evaluate disease activity in UC. We also have expectations for the expression of RPL19 mRNA in serum, which we hope can be developed into a simple and convenient noninvasive marker.

In this experiment, we observed a higher expression of RPL19 mRNA in the inflammatory mucosa of UC patients compared to normal controls. This finding is significant for the clinical differential diagnosis between UC and HC, and it also suggests that RPL19 may play a role in the development of UC. Upon stratifying UC patients by clinical disease activity, we noted that the expression level of RPL19 mRNA in patients within the severe group was significantly higher than in those in the mild and moderate groups. This indicates that RPL19 may be involved in the pathogenesis of severe UC, offering valuable insights for diagnosing and assessing severe UC. However, the potential proinflammatory role of RPL19 in the pathogenesis of UC requires further investigation and validation. Furthermore, we found that the expression level of RPL19 mRNA in the intestinal mucosa of UC patients positively correlated with several disease activity scores, including the modified Mayo score, Mayo score, UCEIS score, and Dublin score. This correlation increased with the severity of the disease, suggesting that RPL19 plays a regulatory role in the progression of UC. The linear relationship between RPL19 expression levels and the clinical severity of UC is significant for assessing clinical disease activity and monitoring disease progression.

According to the Montreal classification, UC patients were divided into three groups: E1, E2, and E3. Our findings revealed that the expression level of RPL19 mRNA was not related to the extent of the lesions, indicating that we cannot predict the extent of UC lesions based solely on RPL19 mRNA expression levels. This study marks the first exploration of the relationship between RPL19 mRNA expression and endoscopic disease activity in UC, assessed in combination with MES, UCEIS, and Dublin scores. We discovered that RPL19 mRNA expression levels in the intestinal mucosa of UC patients were positively and statistically correlated with MES, UCEIS, and Dublin scores. This correlation suggests that RPL19 mRNA expression levels in the intestinal mucosa accurately reflect the degree of intestinal mucosal lesions in UC, providing crucial insights into assessing endoscopic disease activity. Specifically, in the MES and the UCEIS scoring groups, the expression levels of RPL19 mRNA in the intestinal mucosa of patients in the severe group were significantly higher than those in the mild and the moderate groups. However, the differences in expression between the moderate and the mild groups were not consistent. These results confirm that RPL19 mRNA expression levels are closely related to endoscopic disease activity, playing a pivotal role in reflecting this activity in UC. This finding has significant implications for diagnosing severe UC and could serve as an important reference in clinical settings.

In our analysis of serum inflammatory factors from 13 patients, we observed that the level of RPL19 mRNA was negatively correlated with IL‐2 and IL‐4, both critical cytokines in the immune system [28, 29]. IL‐2 primarily functions in the activation and proliferation of T cells, which are essential white blood cells involved in the body’s immune response [30]. IL‐2 also promotes the production of antibodies by B cells and helps regulate the immune response, tilting it towards anti‐inflammatory or allergic reactions. A decrease in IL‐2 impairs the function of regulatory T cells, which are crucial for maintaining immune tolerance. When regulatory T cell function is compromised, the immune system is more prone to attacking the body’s own tissues, including intestinal tissues. This dysfunction can initiate or exacerbate UC [30]. IL‐4 plays a critical role in steering the immune system towards a Th2‐type response, which is anti‐inflammatory and beneficial in the context of UC. A decrease in IL‐4 can cause a shift towards a Th1/Th17‐type response, which is more pro‐inflammatory. This imbalance, characterized by increased pro‐inflammatory Th1 and Th17 cells and decreased regulatory T cells, leads to persistent inflammation in the colon [31, 32]. Chronic inflammation is linked with increased cellular metabolism, DNA damage from oxidative stress, and the release of pro‐inflammatory cytokines and chemokines that encourage tumor growth and survival [33, 34]. This cycle intensifies inflammation in colitis, which over time heightens the risk of progressing to colon cancer [35]. This may explain why fecal RPL19 mRNA expression serves as a prognostic indicator in patients with colorectal cancer, as it likely reflects these underlying inflammatory processes.

Through ROC analysis of RPL19 mRNA expression in the intestinal mucosa of UC patients, we found that RPL19 mRNA levels were more effective than the conventional inflammatory marker CRP in assessing severe UC. This suggests that RPL19 mRNA levels in the intestinal mucosa provide a more accurate reflection of the progression trends in severe UC, offering significant advantages over the traditional CRP index.

While our study offers preliminary insights, it is limited by its small sample size and the fact that it investigates the relationship between RPL19 and UC disease activity solely at the gene level. Future studies should aim to verify these findings at the protein level, expand the sample size, and diversify the detection methods used. Additionally, our data collection was restricted to patients in the active phase of UC; we did not quantitatively analyze RPL19 mRNA expression levels in the intestinal mucosa of patients in remission. Exploring potential differences in RPL19 mRNA expression between patients in the remission stage and those in the active stage would be a valuable avenue for further research.

In conclusion, measuring the expression level of RPL19 mRNA in the intestinal mucosa offers important insights for diagnosing UC and monitoring its progression. We found a positive correlation between RPL19 mRNA expression levels and disease severity, highlighting its utility in assessing severe UC. Therefore, RPL19 mRNA is a promising biomarker for evaluating UC disease activity.

Moreover, the observed changes in inflammatory factors and their potential links with prognostic levels of colon cancer, as noted by prior studies, suggest that RPL19 mRNA could also serve as an indicator for the potential transition from colitis to colitis‐associated colon cancer. However, this study is limited by its cross‐sectional design. The lack of longitudinal, paired samples before and after treatment prevents an assessment of whether changes in mucosal RPL19 mRNA expression correlate with or predict therapeutic efficacy. Further research is necessary to validate this potential and development of RPL19 mRNA as a non‐invasive serum marker and a reliable marker of carcinogenesis in UC patients.

## Author Contributions

All authors contributed to the conceptualization and the writing of this manuscript.

## Funding

This work was supported by the National Natural Science Foundation of China (82270562), the National Natural Science Foundation of Shandong Province (ZR2025MS1260), The Natural Science Foundation of Fujian Province (2023J011709).

## Disclosure

All data presented in the article are the authors’ own research. All authors approved the final version.

## Ethics Statement

Written informed consent was obtained from all the participants who participated in this study. This study was approved by the Clinical Ethics Committee of the Affliated Hospital of Jining Medical University (2021‐09‐c002).

## Conflicts of Interest

The authors declare no conflicts of interest.

## Data Availability

The data that support the findings of this study are available on request from the corresponding author.
